# Constructing High-Performance Solar Cells by Incorporating an A1-A2-Type Polymer Donor as a Guest Material

**DOI:** 10.3390/molecules30244755

**Published:** 2025-12-12

**Authors:** Min Li, Guo Chen, Ai Lan, Sein Chung, Mingming Que, Yongjoon Cho, Bin Huang

**Affiliations:** 1 Ministry of Culture, Sports and Labor, Gannan Health Vocational College, Ganzhou 341000, China; 2 School of Chemistry and Chemical Engineering, Jiangxi University of Science and Technology, 156 Ke Jia Avenue, Ganzhou 341000, Chinabinhuang@jxust.edu.cn (B.H.); 3 Department of Chemical Engineering, Pohang University of Science and Technology (POSTECH), Pohang 37673, Republic of Korea; 4 School of Chemistry and Materials, Gannan Normal University, Ganzhou 341000, China; 5 Department of Physics and Chemistry, Daegu Gyeongbuk Institute of Science and Technology (DGIST), Daegu 42988, Republic of Korea

**Keywords:** polymer donors, A1-A2 type copolymer, guest material, high-performance ternary devices, photostability

## Abstract

Owing to the intramolecular push-pull electron effect between the electron donor (D) unit and electron acceptor (A) unit, the D-A type based polymer donors display outstanding device performance. However, the imperfect energy levels lead to the D-A-type-based polymer device exhibiting high voltage loss. In this study, an A1-A2-type copolymer M1 was developed with 1,3-bis(2-ethylhexyl)-5,7-di(thiophen-2-yl)benzo[1,2-c:4,5-c’]dithiophene-4,8-dione (BDD) as the A1 unit and dithieno[3′,2′:3,4;2″,3″:5,6]benzo[1,2-c][1,2,5]thiadiazole (DTBT) as the A2 unit. Compared with D-A-type-based polymer donor PM6, the A1-A2 type based M1 possesses lower energy levels, broader absorption, and stronger crystallinity. After introducing M1 to the PM6:L8-BO-based system as the guest material, the ternary blend films exhibited exceptional face-on molecular orientation and favorable active-layer morphology, which promotes exciton dissociation and suppresses charge recombination. Consequently, the PM6:M1(5%):L8-BO-based ternary device exhibited an impressive power conversion efficiency (PCE) of 19.70% with simultaneously enhanced photostability, which is superior to the PM6:L8-BO-based binary system. Our work offers an efficient approach to developing high-performance ternary devices by introducing a novel A1-A2 type polymer donors as the guest material.

## 1. Introduction

Polymer solar cells (PSCs) have garnered significant research interest in recent years, owing to their unique advantages such as flexibility, lightweight nature, and stretchability, which make them promising candidates for portable and wearable electronics [1,2,3]. Considerable efforts have been devoted to improving the power conversion efficiency (PCE) of PSCs, with state-of-the-art devices now achieving PCEs exceeding 21% [4,5,6]. Despite these remarkable advancements, the overall performance of PSCs still lags behind that of conventional silicon or emerging perovskite solar cells, primarily due to their relatively large energy loss (*E*_loss_) [7,8,9]. To address this issue and minimize *E*_loss_ in non-fullerene acceptor (NFA)-based PSCs, it is essential to carefully design polymer donors with specific optoelectronic properties. Specifically, polymer donors should not only exhibit a wide bandgap to achieve complementary absorption with the NFAs in the wavelength range of 600–900 nm, but also possess deep-lying highest occupied molecular orbital (HOMO) energy levels. This strategic energy level alignment helps minimize the energy offset between the HOMO of the donor and the lowest unoccupied molecular orbital (LUMO) of the NFA, thereby facilitating more efficient charge separation and transport while reducing open-circuit voltage loss, ultimately leading to enhanced device performance [10,11,12].

D-A-type alternating copolymers are currently among the most widely used high-efficiency polymer donors, because of the intramolecular push–pull electron effect between the electron donor (D) unit and electron acceptor (A) unit, which effectively promotes π-electron delocalization and the formation of quinone structures [13,14]. These effects collectively lead to a reduction in alternating bond length and optical bandgap. Furthermore, the photoinduced intramolecular charge transfer is intrinsically linked to the HOMO energy level of the D unit and the LUMO energy level of the A unit, further contributing to the narrowing of the optical bandgap (*E*_g_^opt^), such as PM6 and D18 [15,16]. While D-A-type polymer donors incorporate various A units, such as 1,3-bis(2-ethylhexyl)-5,7-di(thiophen-2-yl)benzo[1,2-c:4,5-c’]dithiophene-4,8-dione (BDD), dithieno[3′,2′:3,4;2″,3″:5,6]benzo[1,2-c][1,2,5]thiadiazole (DTBT), and quinoxaline (QX), the diversity of these A units remains somewhat limited [17,18]. A significant portion of high-performance polymer donors predominantly utilize benzodithiophene (BDT) as the D unit, which inadvertently restricts the further diversification and development of D-A copolymers [19]. Moreover, the HOMO energy levels of most current D-A alternating polymer donors typically fall within the range of −5.00 to −5.40 eV. This energy range often makes it challenging to achieve a high open-circuit voltage (*V*_oc_) when blended with non-fullerene acceptors (e.g., Y6 and other Y-series derivatives). Therefore, the urgent development of structurally diverse polymer donors with lower HOMO energy levels is crucial.

Deviating from the conventional donor–acceptor (D-A)-based structure, A1-A2-type copolymer donors have gained considerable attention for their unique molecular design and associated advantages. The diverse types and low energy levels of A unit enable the A1-A2-type polymer donors to possess different structures and low-lying energy levels, which helps to enrich the types and choices of polymer donors. Furthermore, A1-A2-type polymers typically exhibit a broader absorption range and enhanced crystallinity compared to their D-A counterparts. This combination of properties results in a complementary absorption profile when paired with narrow-bandgap non-fullerene acceptors (such as Y6 and its derivatives), effectively harnessing more solar photons. In addition, A1-A2 type polymer donors always exhibit good planarity and crystallinity due to excellent π-π conjugated planes of the A unit, and the improved molecular ordering also promotes efficient charge transport, leading to excellent hole mobility, which is crucial for reducing charge recombination and boosting device performance [20]. Despite these distinct advantages in electronic structure and optoelectronic properties, the development of A1-A2 type copolymer donors is hampered by challenges in solubility and active layer morphology control, areas where they currently underperform relative to D-A alternating copolymers. Consequently, the overall device performance of A1-A2 type polymers still lags behind, highlighting a critical direction for future material design and processing optimization.

Among strategies to enhance PCE, ternary strategies offer a simple and effective approach by rationally incorporating a third component to construct D:A1:A2 or D1:D2:A-based architectures to achieve complementary absorption, optimized energy level alignment, and improved blend crystallinity [21,22,23,24,25]. This synergistic effect effectively enhances key photovoltaic parameters, including open-circuit voltage (*V*_oc_), short-circuit current density (*J*_sc_), and fill factor (FF). For high-performance ternary PSCs, the HOMO and LUMO energy levels of the third component should ideally be positioned between those of the host donor and acceptor [26,27]. This cascading energy level structure facilitates efficient charge collection at the electrodes. Furthermore, selecting a guest material with a lower HOMO or higher LUMO level compared to the host system can lead to higher *V*_oc_ values than those of binary PSCs [28,29]. Crucially, the absorption spectrum of the third component must complement those of the host donor and acceptor to maximize photon utilization [30,31,32].

Based on these considerations, we designed and synthesized an A1-A2-type copolymer, utilizing BDD as the A1 unit and DTBT as the A2 unit. Due to the large conjugation plane and strong electron-withdrawing character of both the BDD and DTBT units, the resulting polymer donor, M1, exhibited extended absorption and deeper energy levels compared to the D-A alternating copolymer PM6. After introducing M1 to the PM6:L8-BO-based system, the obtained blend films exhibit a preference for face-on molecular orientation and favorable active-layer morphology to enhance exciton dissociation and suppress charge recombination. As a result, the PM6:M1(5%):L8-BO-based ternary device achieved an impressive PCE as high as 19.70% with *J*_sc_ of 26.96 mA cm^−2^, *V*_oc_ of 0.90 V, and the FF was 81.2%, which is much higher than that of PM6:L8-BO-based binary system (19.02%). This study provides an efficient strategy to construct a highly efficient ternary device by incorporating A1-A2-type copolymer donors with low-lying energy levels, broader absorption range, and strong crystallinity.

## 2. Results and Discussion

**Figure 1:**
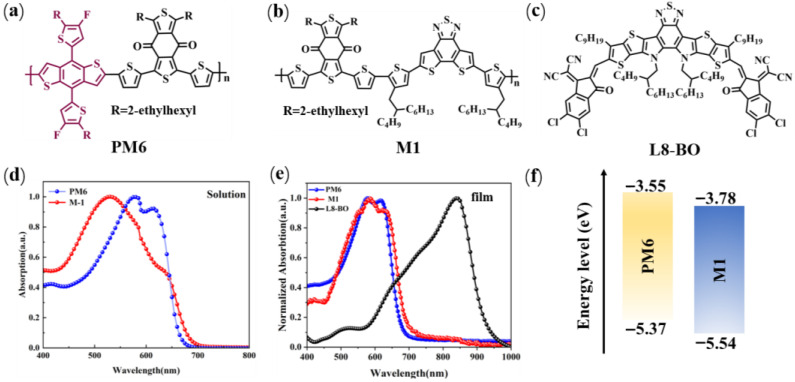
(**a**) The structures of (**a**) PM6, (**b**) M1, and (**c**) L8-BO. UV-vis absorption curve of polymer donors and acceptor (L8-BO) for (**d**) solution and (**e**) film state. (**f**) The energy level of PM6 and M1 was obtained by using Gaussian 16 software at the B3LYP/6-31G(d,p) level based on DFT.

The molecular structures of PM6, M1, and L8-BO (acceptor) used in this study are presented in Figure 1a–c. PM6 was chosen as a reference D-A polymer donor to assess the advantages of the A1-A2-type polymer donor M1. The synthetic route of M1, along with detailed experimental procedures, is provided in the Supporting Information (SI). To confirm the structure of M1, proton nuclear magnetic resonance (^1^H NMR and ^13^C NMR) spectroscopy was performed. As shown in Figures S1 and S2, characteristic signals of the aliphatic hydrocarbon were observed in the range of δ 0.45–2.19 ppm. Furthermore, elemental analysis (EA) was also conducted to certify the structure of M1 based on the ratio of carbon (C), hydrogen (H), nitrogen (N), and sulfur (S) atoms. The obtained ratio of C:H:N:S is 67.89:6.92:2.11:20.64, falling well within the expected stoichiometric ratio, demonstrating the successful incorporation of BDD and DTBT units. In addition, the molecular weight of the polymers was determined using gel permeation chromatography (GPC). The number-average molecular weight (${\overset{-}{M}}_{n}$) of M1 is 13.02 kDa with corresponding polydispersity indices (PDIs) of 3.53 (Figure S3).

The normalized ultraviolet–visible (UV-Vis) absorption spectra of PM6 and M1 are presented in Figure 1d,e, and the related optical data are summarized in Table 1. Both polymers exhibit a clear red shift in their absorption spectra when transitioning from solution to film. As depicted in Figure 1e, the two polymer donor films show broad absorption spectra spanning the range of 300–750 nm, effectively complementing the light absorption of L8-BO. Compared to the PM6 neat film, which has a λ_onset_ of 683 nm and an optical bandgap (*E*_g_^opt^) of 1.82 eV, the M1 neat film exhibits a broader absorption width with a λ_onset_ of 706 nm and an *E*_g_^opt^ of 1.76 eV. This broader absorption in M1 is advantageous for enhancing sunlight utilization and, consequently, could potentially increase the *J*_sc_ of the resulting device.

**Figure 2:**
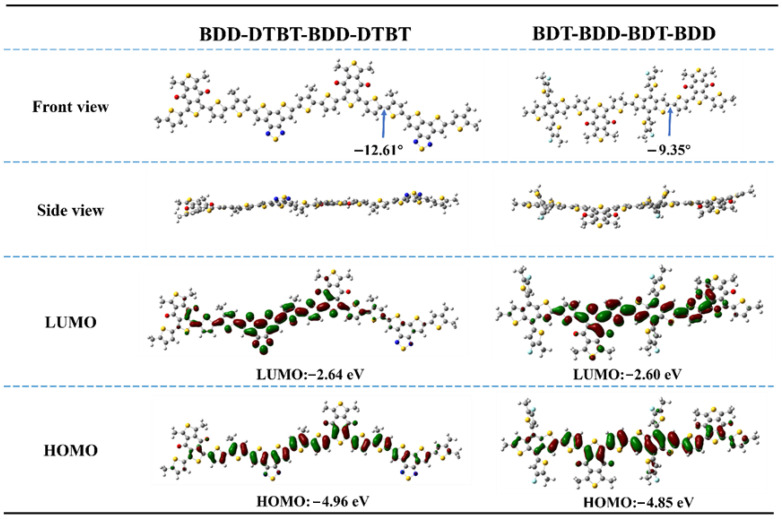
DFT calculation of PM6-based dimer model BDT-BDD-BDT-BDD and M1-based dimer model BDD-DTBT-BDD-DTBT for optimized geometries and energy levels.

To investigate the differences in molecular geometry between two types of molecules with distinct structures, density functional theory (DFT) calculations were performed using Gaussian software at the B3LYP/6-31G(d,p) level of theory. To simplify the DFT calculations, alkyl chains were replaced with methyl groups. The PM6 model was represented by the dimer structure BDT-BDD-BDT-BDD, and the M1 model by BDD-DTBT-BDD-DTBT. As illustrated in Figure 2, the dihedral angle between the BDT and BDD units in PM6 was approximately 9.35°, while the dihedral angle between the DTBT and BDT units in M1 was 12.61°. In addition, we calculated the energy levels of the two polymers using DFT. Compared to the PM6-based model, which exhibited HOMO/LUMO energy levels of −4.86 eV/−2.60 eV, the M1-based model displayed lower energy levels, with HOMO/LUMO energies of −4.95 eV/−2.64 eV. This shift is primarily attributed to the strong electron-withdrawing nature of the DTBT unit.

**Figure 3:**
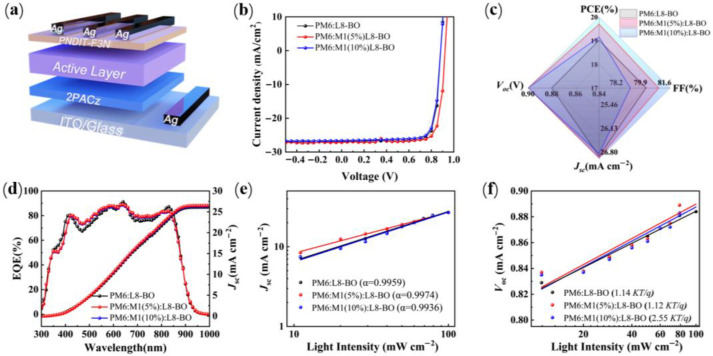
(**a**) Device structure in this work. (**b**) The optimal *J*-*V* characteristics of the PSC devices in this work. (**c**) The distribution of device parameters in this work. (**d**) EQE curves. (**e**) The dependence of *J*_sc_ on the P_light_ for the related device. (**f**) The dependence of V_oc_ on the P_light_ for the related device.

Photovoltaic performance of M1 was studied by the conventional device structure of ITO/2PACz/Polymer donor:L8-BO/PNDIT-F3N/Ag (Figure 3a), where 2-(9H-carbazol-9-yl)ethyl]phosphonic acid (2PACz) as the anode interface layer and poly[(9,9-bis(3′-(N,N-dimethylamino)propyl)-2,7-fluorene)-alt-5,5′-bis(2,2′-thiophene)-2,6-naphthalene-1,4,5,8-tetracaboxylic-N,N′-di(2-ethylhexyl)imide] as the cathode interface layer. The current density–voltage (*J*-*V*) curves of the optimized devices are presented in Figure 3b, and detailed photovoltaic parameters are summarized in Table 2. The PM6:L8-BO-based binary device achieved a reference value as the PCE of 19.06% with a Voc of 0.884 V, a *J*_sc_ of 26.92 mA cm^−2^, and an FF of 80.3%. To thoroughly investigate the impact of M1 on device performance, we fabricated ternary PSCs using PM6:L8-BO as the primary host material. By systematically varying the doping concentration of M1, we observed that a 5% mass ratio of M1 in the PM6:M1(5%):L8-BO-based devices yielded the champion PCE of 19.70% with significant improvements in *J*_sc_ as it reached 26.96 mA cm^−2^, the Voc was 0.901 V, and the FF was 81.2%. In contrast, increasing the doping ratio of M1 to 10% resulted in a decline in performance for the PM6:M1(10%):L8-BO-based devices, with a PCE of 19.16%. The statistics of the solar cell devices are shown in Figure 3c, and the champion PCE value is found in the ternary mixture. The ternary device exhibits superior device performance mainly due to the introduction of M1, enabling the blend films to exhibit exceptional face-on molecular orientation and favorable active-layer morphology, which contribute to the enhancements of *J*_sc_ and FF.

Meanwhile, photostability tests were performed under LED illumination (1 sun) at room temperature to further evaluate the photostability of the related binary device and ternary device. As illustrated in Figure S5, the ternary device demonstrates improved illumination stability compared to binary devices, because the introduction of M1 could potentially mitigate trap-assisted recombination linked to light-induced trap states while helping stabilize the balance between photochemical and morphological properties in the blend system. Additionally, external quantum efficiency (EQE) spectra were tested to explore the effect of different amounts of M1 on the EQE response. As plotted in Figure 3d, the M1-based ternary device offers much higher EQE values in the range of 400 to 850 nm with the integrated short-circuit current (*J*_cal_) is 26.11 mA cm^−2^, 26.53 mA cm^−2^, and 26.35 mA cm^−2^ for PM6:L8-BO, the PM6:M1(5%):L8-BO, and PM6:M1(10%):L8-BO-based devices, respectively, which is closely aligns with the values obtained from the *J*-*V* curves with the errors within 5% (seen Table 2).

The charge recombination behavior was investigated by measuring the dependence of *V*_oc_ and *J*_sc_ under various light intensities (*P*_light_). The relationship between *J*_sc_ and *P*_light_ can be estimated based on the equation *J*_sc_ ∝ (*P*_light_)^α^, where α is the recombination parameter and represents the slope of the curve [33]. If the α value is close to 1, all the free charge carriers in the device can be swept out and efficiently collected by the electrode with negligible bimolecular recombination. As depicted in Figure 3e, the recombination parameter α was determined to be 0.995, 0.997, and 0.994 for the PM6:L8-BO, PM6:M1(5%):L8-BO, and PM6:M1(10%):L8-BO-based devices, indicating that the M1-based ternary device possesses minimal bimolecular recombination than the PM6 binary device. In addition, the trap-assisted recombination was evaluated by examining the dependence of *V*_oc_ on *P*_light_ using the equation of *V*_oc_ ∝ n(KT/q)ln(*P*_light_), where K is the Boltzmann constant, T is the temperature in Kelvin, and q is the elementary charge [34,35]. As shown in Figure 3f, the slope values of PM6:L8-BO, PM6:M1(5%):L8-BO, and PM6:M1(10%):L8-BO-based devices are 1.14, 1.02, and 1.18 kT/q, respectively, indicating that the monomolecular recombination is negligible and bimolecular recombination is dominant in all devices, which explains the enhancement of FF and *J*_sc_. In addition, we also evaluated the hole (*μ*_h_) and electron (*μ*_e_) mobilities through space-charge-limited current (SCLC) measurements to analyze the effect of M1 on the charge-transport properties (Figure S6 and Table S1). The estimated *μ*_h_ and *μ*_e_ is 1.32 × 10^−3^ cm^2^ V^−1^ s^−1^ and 0.92 × 10^−3^ cm^2^ V^−1^ s^−1^ for PM6:L8-BO-based, 1.68 × 10^−3^ cm^2^ V^−1^ s^−1^ and 1.36 × 10^−3^ cm^2^ V^−1^ s^−1^ for PM6:M1(5%):L8-BO-based, 1.59 × 10^−3^ cm^2^ V^−1^ s^−1^ and 1.08 × 10^−3^ cm^2^ V^−1^ s^−1^ for PM6:M1(10%):L8-BO-based devices. The PM6:M1(5%):L8-BO ternary blend displayed the highest mobility value and a more balanced μh/μe ratio (1.23), which explained that the appropriate amount of incorporation of 5%M1 can realize a more efficient exciton generation and charge transfer, resulting in decreasing charge recombination and obtaining higher *J*_sc_ and FF.

**Figure 4:**
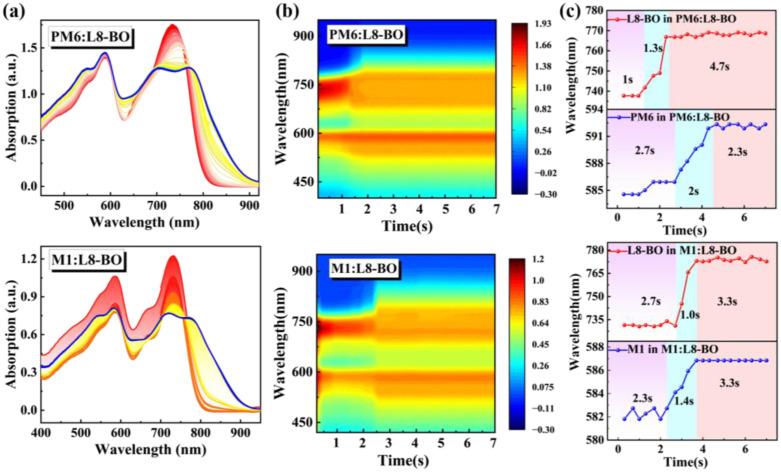
(**a**) In situ UV-vis absorption of PM6:L8-BO and M1:L8-BO blend films. (**b**) Two-dimensional time-resolved UV-vis absorption spectra of PM6:L8-BO and M1:L8-BO blend films. (**c**) The peak position evolution as a function of time for PM6:L8-BO and M1:L8-BO blend films.

The crystallization kinetics of the blend film were analyzed employing in situ UV-vis absorption spectroscopy. The two-dimensional time-resolved UV-vis absorption spectra and contour plots of PM6:L8-BO and M1:L8-BO are presented in Figure 4a,b, respectively. Notably, in comparison with the 29 nm redshift absorption of acceptor from 737 to 766 nm in PM6-based blend film, the acceptor in M1-based blend film displays a more significant 41 nm redshift absorption, ranging from 731 nm to 772 nm, which is due to the intense intermolecular interactions and exceptional miscibility between M1 and L8-BO. This observation is further substantiated in Figure 4c to depict the temporal evolution of peak positions in the blend film. The crystallization time manifested by the second stage of L8-BO in M1:L8-BO blend film is a mere 1.0 s, distinctly shorter than the 1.3 s observed for the PM6:L8-BO blend film. This rapid crystallization time signifies a more pronounced aggregation between M1 and L8-BO molecules, facilitating the formation of well-ordered molecular structures and promoting the optimization of excellent morphology.

**Figure 5:**
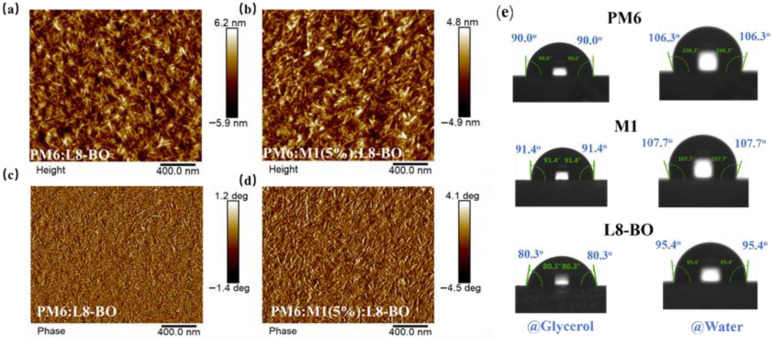
Height images of (**a**) PM6:L8-BO and (**b**) PM6:M1(5%):L8-BO-based blend films. Phase images of (**c**) PM6:L8-BO and (**d**) PM6:M1(5%):L8-BO-based blend films. (**e**) Contact angle measurement of PM6, M1, and L8-BO.

Atomic force microscopy (AFM) was employed to analyze the morphology of the blend films. As shown in Figure 5a,b, the PM6:L8-BO and PM6:M1(5%):L8-BO-based blend films exhibited root-mean-square (RMS) surface roughnesses of 1.58 nm and 1.74 nm, respectively. This indicates a smoother and more uniform morphology for the PM6:M1(5%):L8-BO-based blend films. Moreover, the PM6:M1(5%):L8-BO-based blend films displayed both a characteristic fibrillar network and nanoscale phase separation (Figure 5c,d), potentially contributing to the enhanced *J*_sc_ and FF observed in the corresponding devices. In addition, contact angle of the neat films was performed to evaluate the miscibility of the polymer donor and acceptor according to the Flory–Huggins interaction parameter χ, which can be obtained from the formula of χ_donor,acceptor_ = K(γ_donor_^1/2^ − γ_acceptor_^1/2^)^2^ (χ_donor,acceptor_) is the Flory–Huggins interaction parameter between the donor and acceptor, and K is a constant) [36,37]. As shown in Figure 5e and detailed in Table S2, glycerol and deionized water were used as the calibration liquids. The calculated value of the surface energy (γ) is 21.42 mN/m for PM6, 20.59 mN/m for M1, and 27.45 mN/m for L8-BO, leading to the χ_donor,acceptor_ being 0.36 k for PM6:L8-BO-based blend and 0.48 k for M1:L8-BO-based blend, which can be expected that controlling the amount of M1 to the PM6:L8-BO-based system helps to obtain desirable phase separation in the ternary blend films. These findings agree well with the AFM results.

**Figure 6:**
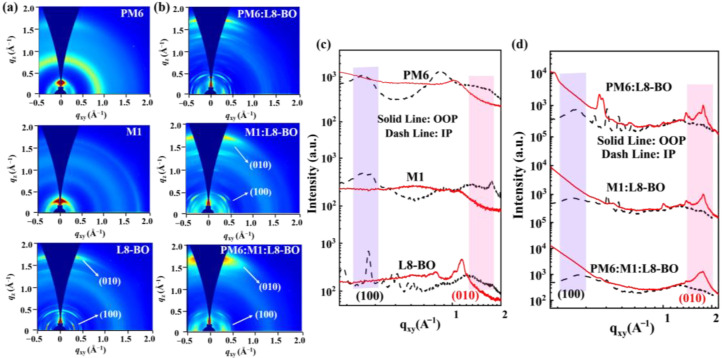
Two-dimensional GIWAXS patterns of (**a**) neat film and (**b**) blend films. Corresponding 1D line profiles in the IP and OOP direction of (**c**) neat films and (**d**) blend films.

Grazing incident wide-angle X-ray scattering (GIWAXS) analysis was performed to explore the molecular orientation and crystallinity of the thin and blend films. As displayed in the 2D GIWAXS patterns (Figure 6) and corresponding 1D intensity profiles (as summarized in Table S3), all the films exhibit a predominant face-on orientation with well-defined π-π stacking diffraction peaks ((010) peak) in the out-of-plane (OOP) direction. Compared to the PM6 thin film with the π-π stacking distance of 3.72 Å and crystal coherence length (CCL) of 14.66 Å, the M1-based neat film exhibits well-defined diffraction peak signals and strong diffraction intensities with a π-π stacking distance of 3.63 Å and a CCL of 18.46 Å in the OOP direction, suggesting that M1 possesses stronger crystallinity than PM6. After combining the M1 to PM6:L8-BO-based blend film, the M1-based ternary blend films displayed predominant face-on molecular packing orientation with well-defined π-π stacking diffraction peaks in the OOP direction and the lamellar stacking peaks in the in-plane (IP) direction. With the proportion of M1 increased from 5% to 10%, the ternary blend films exhibit an increased coherence length in the (100) peak along the IP direction as the CCL and d-spacing is 19.78 Å and 21.52 Å for PM6:L8-BO-based blend film, 20.06 Å and 22.85 Å for PM6:M1(5%):L8-BO-based blend film, and 51.43 Å and 20.94 Å for PM6:M1(10%):L8-BO-based blend film, respectively, indicating that after introducing M1 as the third component, the ternary devices have more orderly molecular arrange, lead to improving carrier transport and diminishing charge recombination, hence beneficial to acquire enhanced FF and PCE.

## 3. Materials and Methods

### 3.1. Materials

All reactions and manipulations were performed under an argon atmosphere. The starting materials were obtained from commercial suppliers and utilized without additional purification. Chloroform, Chlorobenzene, Ag (99.999%), and other materials were purchased from Alfa (Tewksbury, MA, USA), Aldrich (St. Louis, MO, USA, used without further purification). Indium-tin oxide (ITO) glass was purchased from Delta Technologies Limited (Loveland, CO, USA). PACz (Baytron PAl4083) was obtained from Bayer Inc. (Leverkusen, North Rhine-Westphalia, Germany). DTBT-2Br monomer and BDD-2Sn monomer were purchased from SunaTech Inc. (Daejeon, Republic of Korea). and Solarmer Materials Inc (El Monte, CA, USA). Pd_2_(dba)_3_, Pd(PPh_3_)_4_, and Tris-(2-methoxyphenyl)-phosphine (P(2-Meoph)_3_) were obtained from J&K (Beijing, China). L8-BO was purchased from Nanjing Zhiyan Technology Co., Ltd. (Nanjing, China).

### 3.2. Synthetic Procedures

Synthesis of M1: In a round-bottom flask, BDD-2Sn (0.0500 g, 1 mmol), DTBT-2Br (0.0490 g, 1 mmol), Pd_2_(dba)_3_ (0.0010 g, 0.02 mmol), P(2-Meoph)_3_ (0.0013 g, 0.08 mmol) were dissolved in ultra dry toluene (15.0 mL). The mixture was deoxygenated with nitrogen 3 times and stirred at 115 °C for 48 h. After cooling to room temperature, the mixture was dropped into methanol (50 mL) and filtered. The collected crude product was Soxhlet extracted with acetone and chloroform. Finally, the chloroform fraction was concentrated and dropped into methanol (50 mL), filtered, and dried under vacuum to obtain M1 (yield 0.075 g). ^1^H NMR (400 MHz, Chloroform-*d*) δ 7.26 (s, 1H), 2.19–0.45 (m, 27H), 0.03 (d, *J* = 27.9 Hz, 4H). Elemental Analysis calculated for M1: C, 67.89; H, 6.92; N, 2.11; S, 20.64. The theoretical EA values for M1: C, 67.41; H, 6.85; N, 2.07; S, 21.31.

### 3.3. Device Fabrication and Characterizations

The PSCs were fabricated with a structure of glass/ITO/2PACz/Active layer /PNDIT-F3N-Br/Ag. The ITO-coated glasses were ultrasonically precleaned with detergent, deionized water, acetone, and 2-propanol for 30 min each and dried by a nitrogen blow. The ITO glasses were treated with UV-ozone for 20 min before use. 2PACz was spin-coated onto the ITO substrate and then annealed in an oven for 5 min at 100 °C. Then the device was transferred to a nitrogen glove box. The active layer was spin-coated from a 14.0 mg mL^−1^ solution dissolved in chloroform (Polymer donor:L8-BO = 1:1.2, 0.3% *v*/*v* DIO) at varied spinning speeds for 30 s to form an active layer. Subsequently, an ethanol (EtOH) solution of PNDIT-F3N with a small amount of acetic acid at a concentration of 0.5 mg ml^−1^ was deposited atop the active layer at 3000 r.p.m. for 30 s to afford a PNDIT-F3N cathode buffer layer with a thickness of about 10 nm. Finally, the top Ag electrode was deposited over the active layer by thermal evaporation under a vacuum chamber to accomplish the device fabrication. The effective area of one cell was 0.04 cm^2^. The current-voltage (*J*-*V*) characteristics were measured by a Keithley 2400 Source Meter (Cleveland, OH, USA) under simulated solar light (100 mW/cm^2^, AM 1.5 G, Abet Solar Simulator Sun2000 (Milford, CT, USA)). The incident photon-to-electron conversion efficiency (IPCE) spectra were detected on an IPCE measuring system (Oriel Cornerstone 2601/4 m monochromator equipped with Oriel 70613NS QTH lamp (Stratford, CT, USA)). All the measurements were performed at room temperature under a nitrogen glove box.

### 3.4. Characterizations

Details of *J-V*, UV-vis absorption spectra (UV), cyclic voltammetry (CV), and atomic force microscope (AFM) measurements are available in the Supporting Information. Origin 2022 and Gwyddion (64bit) v3.6 were used as software for data analysis and image processing, respectively.

## 4. Conclusions

In conclusion, we synthesized an A1-A2 type copolymer, M1, utilizing BDD as the A1 unit and DTBT as the A2 unit. The developed M1 exhibited broader absorption, lower energy levels, and enhanced crystallinity than that of PM6. After incorporating the M1 into the PM6:L8-BO-based system, the ternary blend films exhibited a well-defined fibrillar morphology and face-on molecular orientation to allow balanced exciton and free charge transport and to suppress recombination. Ultimately, the PM6:M1(5%):L8-BO-based ternary device achieved a PCE of 19.70% with superior photostability, surpassing that of the PM6:L8-BO-based binary device. Our work demonstrated that the ternary strategy of incorporating A1-A2 type polymer donor with deep energy levels, broad absorption range, and strong crystallinity is a highly effective approach for the fabrication of efficient and stable PSCs.

## Figures and Tables

**Table 1:** Physicochemical parameters of PM6 and M1.

Polymers	λ_max_ [nm] ^a^	λ_onset_ [nm] ^b^	$E_{g}^{opt}$ [eV] ^c^	HOMO [eV] ^d^	LUMO [eV] ^e^
PM6	585.8	683	1.82	−5.37	−3.55
M1	617.6	706	1.76	−5.54	−3.78

^a,b^ Obtained from UV-*V*is absorption in the film state. ^c^ Calculated from the empirical formula: E_g_ = 1240/λ_onset_. ^d^ Using the cyclic voltammetry (CV) method, E_HOMO_ = −(4.40 + E_ox_) (eV). ^e^ Calculated from the equation E_LUMO_ = E_HOMO_ + E_g_.

**Table 2:** Photovoltaic parameters under various polymer donors under AM 1.5 G illumination at 100 mW cm^−2^.

Active Layers	*V*_oc_ (V)	*J*_sc_ (mA cm^−2^)	*J*_cal_ (mA cm^−2^) ^a^	FF (%)	PCE (%) ^b^
PM6:L8-BO	0.88	26.92	26.11	80.3	19.02 (19.01 ± 0.01)
PM6:M1(5%):L8-BO	0.90	26.96	26.53	81.2	19.70 (19.26 ± 0.34)
PM6:M1(10%):L8-BO	0.90	26.85	26.35	79.3	19.16 (18.89 ± 0.27)

^a^ Integrated from the EQE curves. ^b^ Average parameters are derived from over 10 independent cells.

## Data Availability

The original contributions presented in the study are included in the article/Supplementary Material; further inquiries can be directed to the corresponding authors.

## Supplementary Materials

The following supporting information can be downloaded at: https://www.mdpi.com/article/10.3390/molecules30244755/s1. Figure S1: ^1^H NMR spectra of M1 in CDCl_3_; Figure S2: ^13^C NMR spectra of M1 in CDCl_3_; Figure S3: GPC test pattern of M1; Figure S4: cyclic voltammetry (CV) measurement of the related polymers; Figure S5: Photostability of the related device; Figure S6: The *μ*_h_ and *μ*_e_ for the blend films; Table S1: Electron and hole mobilities obtained by SCLC measurement; Table S2: Surface tension and the Flory−Huggins interaction parameter of neat film of polymer donors and L8-BO acceptor; Table S3: Summarized parameters for the ordering structures of neat films and blend films.
