# Supplementary material for: Constructing High-Performance Solar Cells by Incorporating an A1-A2-Type Polymer Donor as a Guest Material
Source: Molecules. 2025 Dec 12;30(24):4755. doi: 10.3390/molecules30244755 (PMC12735647; doi:10.3390/molecules30244755)
Supplement: Supplementary file 1 [file molecules-30-04755-s001.zip › molecules-4008152-supplementary.pdf]

Supporting Information

Constructing High-Performance Solar Cells by Incorporating an A1-A2 Type Polymer Donor as a Guest Material

Min Li[1*], Guo Chen[2], Ai Lan[2], Sein Chung[4], Mingming Que[5*], Yongjoon Cho[3*], and Bin Huang[2]

[1] Ministry of Culture, Sports and Labor; Jiangxi Gannan Health Vocational College, Ganzhou, 341000, China.

[2] School of Chemistry and Chemical Engineering, Jiangxi University of Science and Technology, 156 Ke Jia Avenue, Ganzhou, 341000, China.

[3] Department of Physics and Chemistry, Daegu Gyeongbuk Institute of Science and Technology (DGIST), Daegu, 42988, Republic of Korea.

[4] Department of Chemical Engineering, Pohang University of Science and Technology (POSTECH), Pohang 37673, Republic of Korea.

[5] School of Chemistry and Materials, Gannan Normal University, Ganzhou 341000, 341000, China.

Corresponding author: Dr. Min Li, Dr. Mingming Que, and Dr. Yongjoon Cho.

E-mail: lm0908@126.com (M. Li), 2700002@gnnu.edu.cn (M. Que), and yongjoon.cho@northwestern.edu (Y. Cho).

Contents

1. **General Information**

2. **Nuclear magnetic resonance (NMR) and gel permeation chromatography (GPC) measurement**

3. **Optical characterizations**

4. **Electrochemical characterizations.**

5. **AFM characterizations.**

6. **GIWAXS measurements**

7. **Device Fabrication and Characterizations**

8. **Results and Discussion.**

General Information

All reactions and manipulations were operated under argon-atmosphere, and all the starting materials were purchased from commercial suppliers and used without further purification. Chloroform, Chlorobenzene, Ag (99.999%) and other materials were purchased from Alfa, Aldrich (used without further purification). Indium-tin oxide (ITO) glass was purchased from Delta Technologies Limited. PACz (Baytron PAl4083) was obtained from Bayer Inc. DTBT-2Br and BDD-2Br were purchased from SunaTech Inc and Solarmer Materials Inc. $Pd_2(dba)_3$, $Pd(PPh_3)_4$, and $P(2\text{-}Meoph)_3$ were obtained from J&K. L8-BO was purchased from Nanjing Zhiyan Technology Co.， Ltd.

Nuclear magnetic resonance (NMR) and gel permeation chromatography (GPC) measurement

^{1}H NMR spectra of the polymers were recorded on a Bruker AVANCE III HD 400 MHz spectrometer using deuterated $CDCl_3$ as solvent and tetramethylsilane (TMS) as an internal standard. The average number (M_n) and weight (M_w) molecular weight and PDI of the copolymer products were determined by Waters 2410 gel permeation chromatography (GPC) at 100 °C using 1,2,4-trichlorobenzene as an eluent.

Optical characterizations

UV-vis absorption spectra were recorded on a Perkin Elmer Lambda 750 spectrophotometer. All film samples were spin-cast on quartz slice substrates. Solution UV-*vis* absorption spectra at elevated temperatures also were collected on a Perkin Elmer Lambda 750 Spectrophotometer.

Electrochemical characterizations.

Cyclic voltammetry (CV) was performed by a Zahner IM6e electrochemical work station, using Ag/AgCl as the reference electrode, a Pt plate as the counter electrode, and a glassy carbon as the working electrode. Polymers were drop-cast onto the electrode from chloroform

solutions to form thin films. 0.1 mol L^{-1} tetrabutylammonium hexafluorophosphate in anhydrous acetonitrile was used as the supporting electrolyte. The scan rate was 0.05 V s^{-1}. The E_{HOMO} and E_{LUMO} are calculated as referring to the equation (1) and (2).

$$E_{HOMO}=-(E_{ox}+4.4)\ eV\ (1),$$

$$E_{LUMO}=-(E_{red}+4.4)\ eV\ (2).$$

AFM characterizations.

The specimen for AFM measurements was prepared using the same procedures those for fabricating devices but without MoO_3/Ag on top of the active layer.

GIWAXS measurement

The GIWAXS measurement was carried out at the 3C SAXS-I and 9A U-SAXS beamline of the Pohang Accelerator Laboratory in Korea. The X-rays coming from the in-vacuum undulator (IVU) were monochromatic (wavelength λ=1.10994 Å) using a double crystal monochromator and focused both horizontally and vertically (450 (H) x 60 (V) um^2 in FWHM @ the sample position) using K-B type mirrors. The grazing incidence wide-angle X-ray scattering (GIWAXS) sample stage was equipped with a 7-axis motorized stage for the fine alignment of the sample, and the incidence angles of the X-ray beam were set to be 0.11°-0.13° for the neat and blend films. The GIWAXS patterns were recorded with a 2D CCD detector (Rayo nix SX165) and an X-ray irradiation time within 100 s, dependent on the saturation level of the detector. Diffraction angles were calibrated using a sucrose standard (monoclinic, P21, a=10.8631Å, b=8.7044Å, c=7.7624Å, and b=102.938Å) and the sample-to-detector distance was ~231 mm.

Device Fabrication and Characterizations

The PSCs were fabricated with a structure of glass/ITO/2PACz/Active layer /PNDIT-F3N-Br/Ag. The ITO-coated glasses were ultrasonic precleaned with detergent, deionized water, acetone and 2-propanol for 30min each and dried by a nitrogen blow. The ITO glasses were treated with UV-ozone for 20 min before use. 2PACz was spin-coated onto the ITO substrate and then

annealed in an oven for 5min at 100 °C. Then the device was transferred to a nitrogen glove box. The active layer was spin-coated from 14.0 mg mL^{-1} solution dissolved in chloroform (Polymer donor:L8-BO=1:1.2, 0.3% v/v DIO) at varied spinning speed for 30 s to form an active layer. Subsequently, ethanol (EtOH) solution of PNDIT-F3N with a small amount of acetic acid at a concentration of 0.5 mg ml^{-1} was deposited atop the active layer at 3,000 r.p.m. for 30 s to afford a PNDIT-F3N cathode buffer layer with thickness of about 10 nm. Finally, top Ag electrode was deposited over the active layer by thermal evaporation under a vacuum chamber to accomplish the device fabrication. The effective area of one cell was 0.04 cm^2. The current-voltage (J-V) characteristics were measured by a Keithley 2400 Source Meter under simulated solar light (100 mW/cm^2, AM 1.5 G, Abet Solar Simulator Sun2000). The incident photon-to-electron conversion efficiency (IPCE) spectra were detected on an IPCE measuring system (Oriel Cornerstone 2601/4 m monochromator equipped with Oriel 70613NS QTH lamp). All the measurements were performed at room temperature under nitrogen glove box.

Results and Discussion

Synthetic procedures

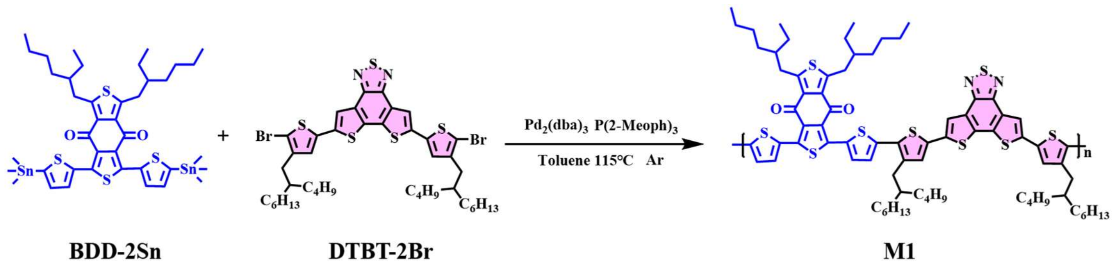

Synthesis of M1: In a round bottom flask, BDD-2Sn (0.0500 g, 1 mmol), DTBT-2Br (0.0490 g, 1 mmol), $Pd_2(dba)_3$ (0.0010 g, 0.02 mmol), P(2-Meoph)$_3$ (0.0013 g, 0.08 mmol) were dissolved in ultra dry toluene (15.0 mL). The mixture was deoxygenated with nitrogen for 3 times and stirred at 115℃ for 48h. After cooling to room temperature, the mixture was dropped into methanol (50 mL) and filtered. The collected crude product was Soxhlet extracted with acetone and chloroform. Finally, the chloroform fraction was concentrated and dropped into methanol (50 mL), after filtered and dried under vacuum to obtain M1 (yield 0.075 g). ^{1}H NMR (400 MHz, Chloroform-*d*) δ 7.26 (s, 1H), 2.19-0.45 (m, 27H), 0.03 (d, *J* = 27.9 Hz, 4H). Elemental Analysis calculated for M1: C, 67.89; H, 6.92; N, 2.11; S ,20.64. The theoretical EA values for M1: C, 67.41; H, 6.85; N, 2.07; S ,21.31.

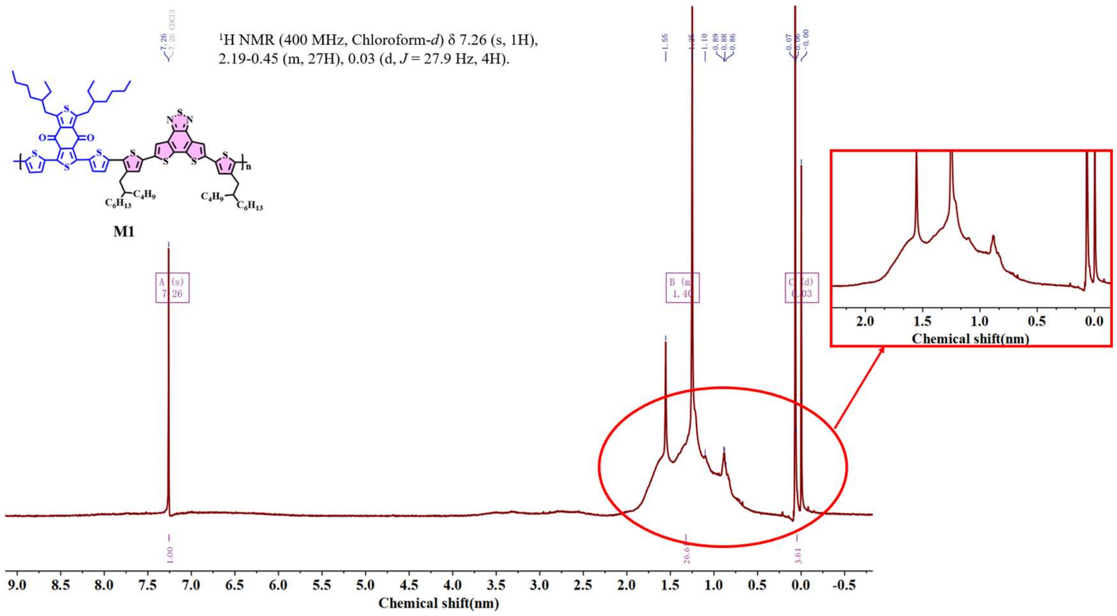

Figure S1. ^{1}H NMR spectra of M1 in $CDCl_3$.

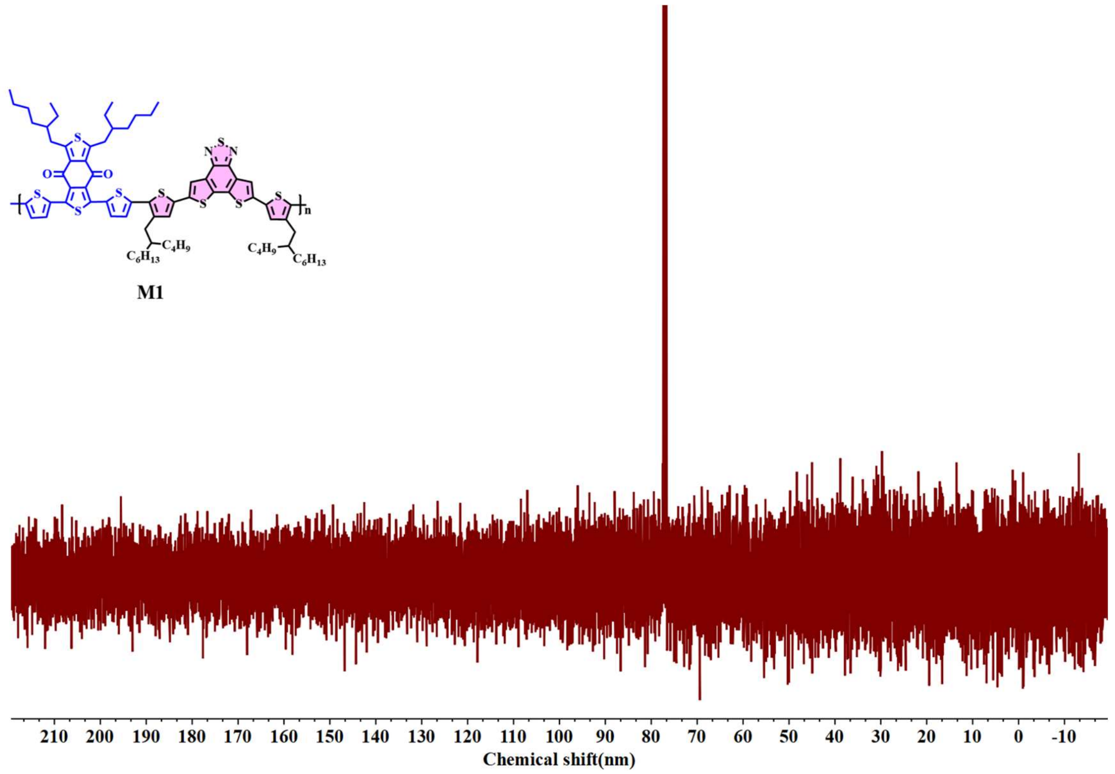

Figure S2. ^{13}C NMR spectra of M1 in $CDCl_3$.

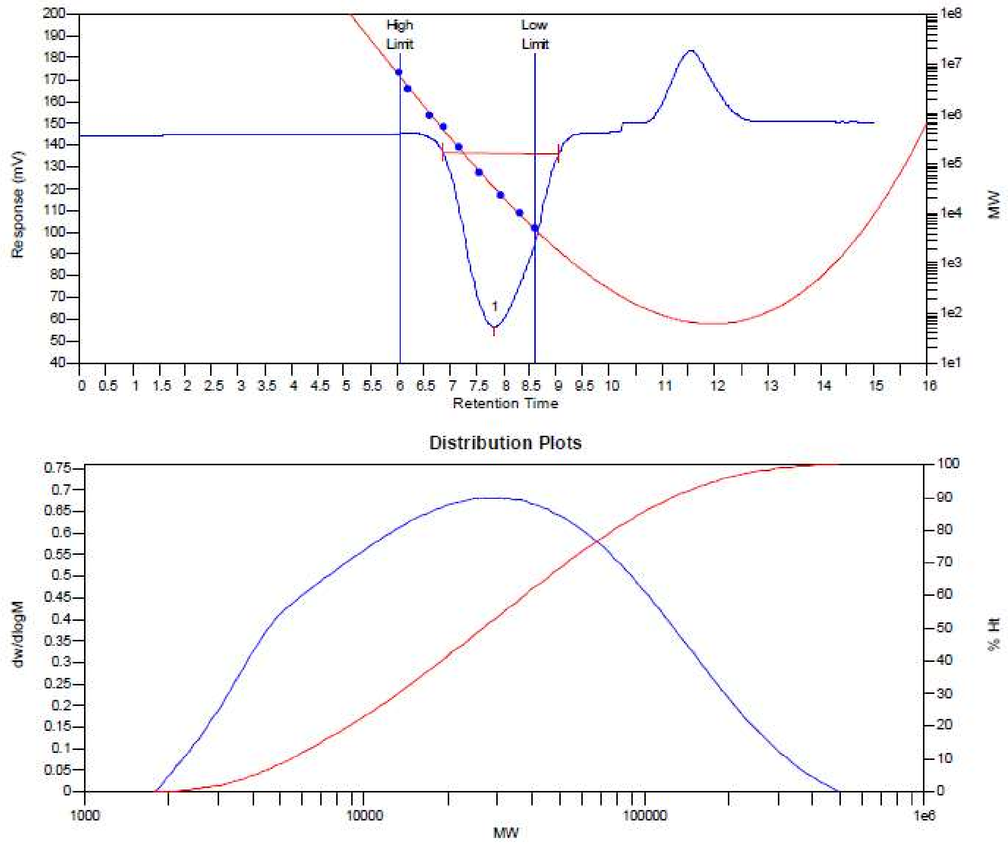

MW Averages

Peak No	Mp	Mn	Mw	Mz	Mz+1	Mv	PD
1	33247	13023	46096	120343	196993	38446	3.53958

Processed Peaks

Peak No	Name	Start RT (mins)	Max RT (mins)	End RT (mins)	Pk Height (mV)	% Height	Area (mV.secs)	% Area
1		6.87	7.83	9.05	-79.2985	0	6065.8	100

Figure S3. GPC test pattern of M1.

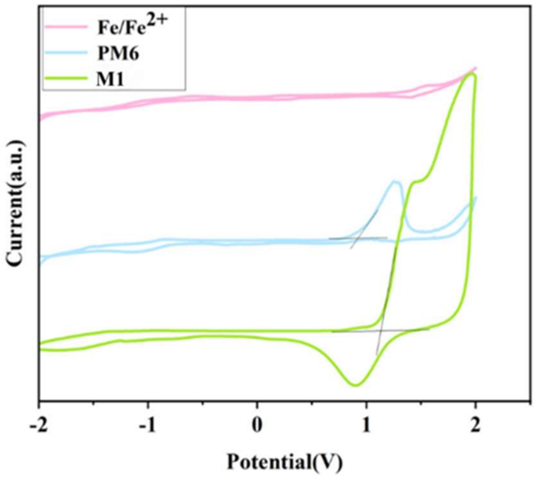

Figure S4. Cyclic voltammetry (CV) measurement of the related polymers.

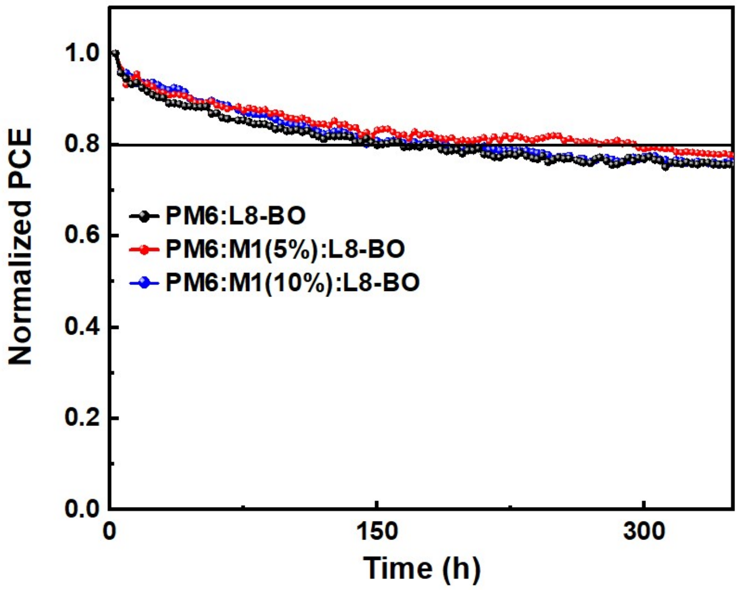

Figure S5. Photostability of the related device.

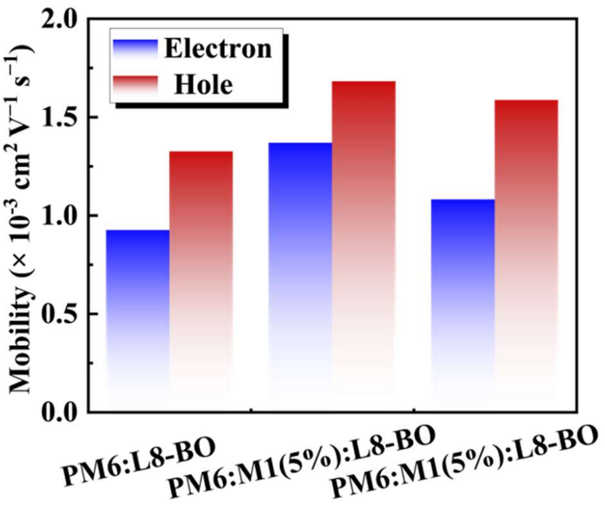

Figure S6. The μ_h and μ_e for the blend films.

Table S1. Electron and hole mobilities obtained by SCLC measurement.

Test film	μ_h [cm^2 V^{-1} s^{-1}]	μ_e [cm^2 V^{-1} s^{-1}]	μ_h/μ_e
**PM6:L8-BO**	1.32×10-3	0.92×10-3	1.45
**PM6:M1(5%):L8-BO**	1.68×10-3	1.36×10-3	1.12
**PM6:M1(10%):L8-BO**	1.59×10-3	1.08×10-3	1.46

Table S2. Surface tension and the Flory−Huggins interaction parameter of neat film of polymer donors and L8-BO acceptor.

Film	θ_{water} [°]	$\theta_{glyceron}$ [°]	γ [mN m^{-1}]	Blend film	χ/K(γ_D^{-2}-γ_A^{-2})^2
**PM6**	106.3	90.0	21.42	PM6:L8-BO	0.36 K
**M1**	107.7	91.4	20.59	M1:L8-BO	0.48 K
**L8-BO**	95.4	80.3	27.45	/	/

Tables S3. Summarized parameters for the ordering structures of neat films and blend films.

Samples	π-π stacking (010) along OOP direction				Lamella stacking (100) along IP direction			
	q ($Å^{-1}$)	*d*-spacing (Å)	FWHM ($Å^{-1}$)	CCL (Å)	q ($Å^{-1}$)	d-spacing (Å)	FWHM ($Å^{-1}$)	CCL (Å)
**PM6**	1.69	3.72	0.39	14.66	0.28	22.36	0.27	20.64
**M1**	1.73	3.63	0.31	18.46	0.28	22.68	0.29	19.71
**L8-BO**	1.72	3.65	0.10	57.21	0.30	20.81	0.30	18.67
**PM6: L8-BO**	1.77	3.56	0.07	79.18	0.29	21.52	0.29	19.78
**PM6:M1(5%): L8-BO**	1.78	3.53	0.13	45.09	0.28	22.85	0.28	20.06
**PM6:M1(10%): L8-BO**	1.78	3.53	0.14	40.90	0.3	20.94	0.11	51.43
